# Supplementary figures and images for: Social-media based Health Education plus Exercise Programme (SHEEP) to improve muscle function among community-dwelling young-old adults with possible sarcopenia in China: A study protocol for intervention development
Source: PLoS One. 2024 Mar 28;19(3):e0286490. doi: 10.1371/journal.pone.0286490 (PMC10977808; doi:10.1371/journal.pone.0286490)

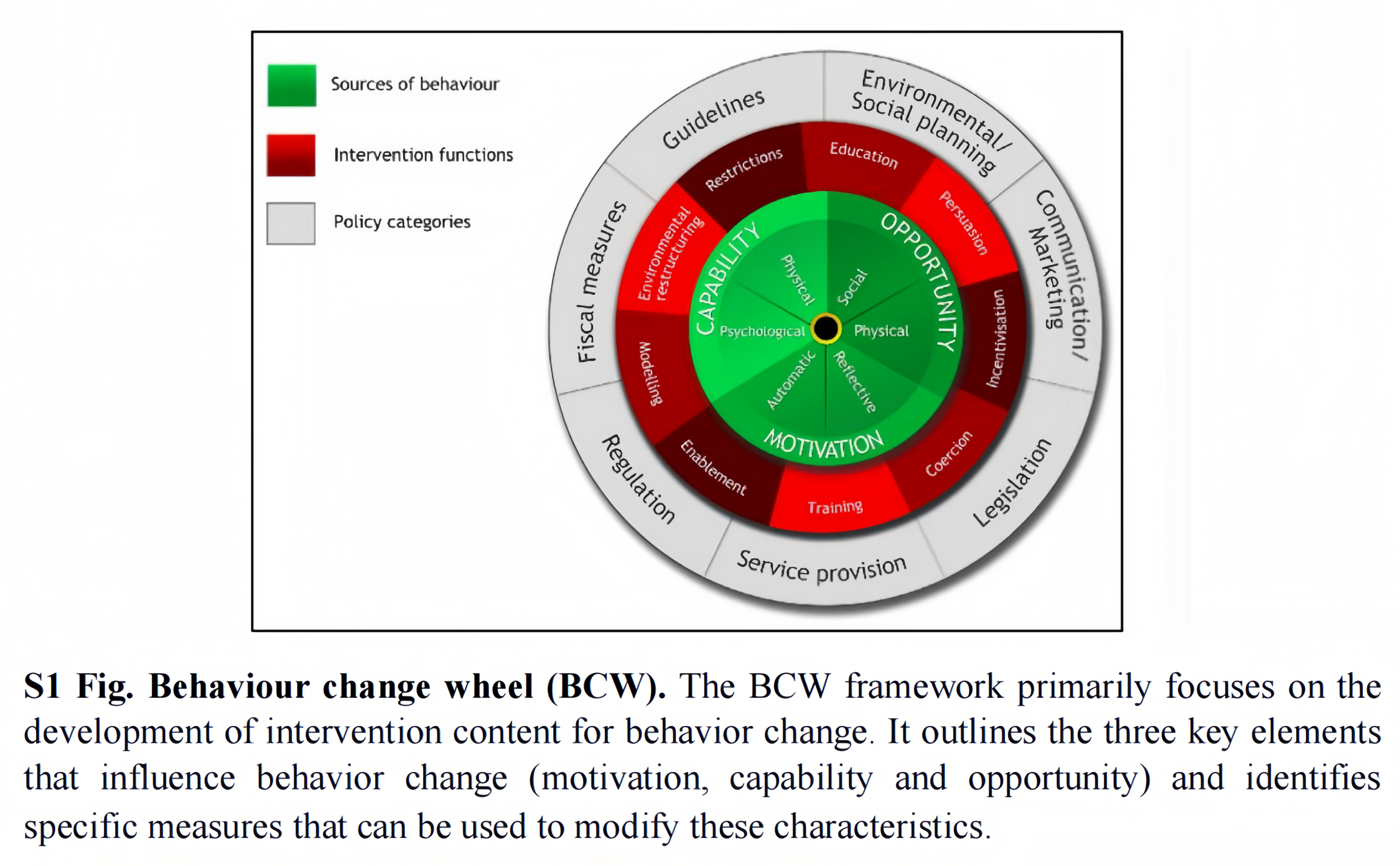

Supplement: S1 Fig — (TIF) [file pone.0286490.s001.tif]

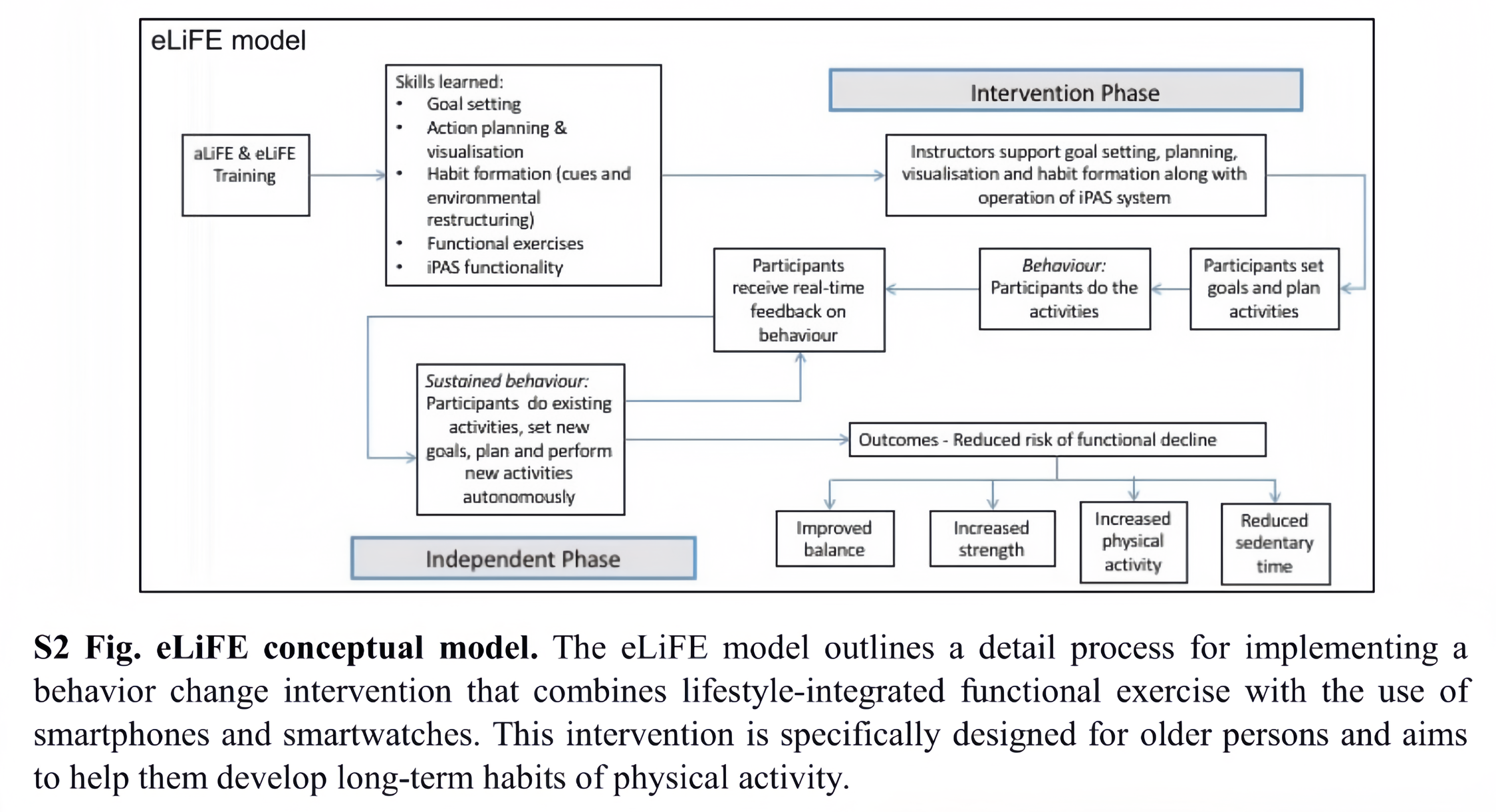

Supplement: S2 Fig — (TIF) [file pone.0286490.s002.tif]

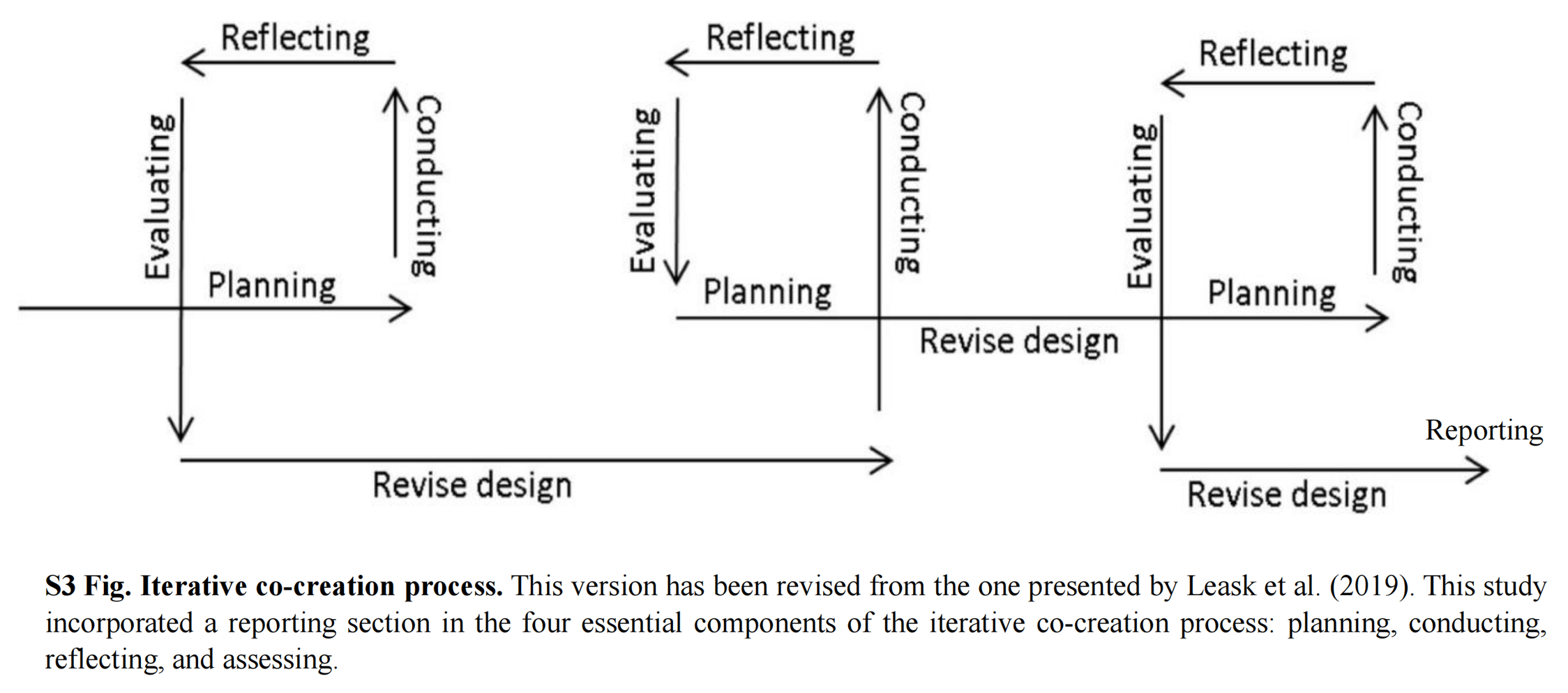

Supplement: S3 Fig — (TIF) [file pone.0286490.s003.tif]
