# Supplementary material for: Social-media based Health Education plus Exercise Programme (SHEEP) to improve muscle function among community-dwelling young-old adults with possible sarcopenia in China: A study protocol for intervention development
Source: PLoS One. 2024 Mar 28;19(3):e0286490. doi: 10.1371/journal.pone.0286490 (PMC10977808; doi:10.1371/journal.pone.0286490)
Supplement: Attachment — Submitted filename: 2023-07-21 Response to Reviewers.docx [file pone.0286490.s005.docx]

**Response to comments**

**Dear Editor and Reviewers:**

Manuscript ID number: PONE-D-23-14790

Title: Social-media based Health Education plus Exercise Programme (SHEEP) to improve muscle function among young-old adults with possible sarcopenia in the community: a study protocol for intervention development

Thank you so much for your insightful feedback on our manuscript. We have revised and responded to each comment in accordance with your suggestion, as demonstrated below.

| **Journal requirements, editor comments and reviewers’ comments** | **Response** | **Page** |
| --- | --- | --- |
| Journal Requirements | |  |
| 1. Please ensure that your manuscript meets PLOS ONE's style requirements, including those for file naming. | We have modified our manuscript according to the requirements of two documents, PLOSOne_formatting_sample_main_body and PLOSOne_formatting_sample_title_authors_affiliations. | -- |
| 2. We noticed you have some minor occurrence of overlapping text with the following previous publication(s), which needs to be addressed.   In your revision ensure you cite all your sources (including your own works), and quote or rephrase any duplicated text outside the methods section. Further consideration is dependent on these concerns being addressed. | We have cited all appropriate sources, including our own publications, and have rephrased or removed any duplicated text. | 5 |
| 3. Please provide additional details regarding participant consent. In the ethics statement in the Methods and online submission information, please ensure that you have specified (1) whether consent was informed and (2) what type you obtained (for instance, written or verbal, and if verbal, how it was documented and witnessed). If your study included minors, state whether you obtained consent from parents or guardians. If the need for consent was waived by the ethics committee, please include this information.  If you are reporting a retrospective study of medical records or archived samples, please ensure that you have discussed whether all data were fully anonymized before you accessed them and/or whether the IRB or ethics committee waived the requirement for informed consent. If patients provided informed written consent to have data from their medical records used in research, please include this information. | We have added a brief explanation in “recruitment, consent and withdrawal” section about when and how informed consent will be obtained from participants, as well as how it should be recorded if consent is given verbally. | 8-9 |
| 4. We note that the grant information you provided in the ‘Funding Information’ and ‘Financial Disclosure’ sections do not match.  When you resubmit, please ensure that you provide the correct grant numbers for the awards you received for your study in the ‘Funding Information’ section. | We have modified the ‘Funding Information’ and ‘Financial Disclosure’ sections as required. | 1-2 |
| 5. Your ethics statement should only appear in the Methods section of your manuscript. If your ethics statement is written in any section besides the Methods, please move it to the Methods section and delete it from any other section. Please ensure that your ethics statement is included in your manuscript, as the ethics statement entered into the online submission form will not be published alongside your manuscript. | We have moved the ethics statement to the Methods section. | 9 |
| 6. Please include a separate caption for each figure in your manuscript. | We have captioned all the figures and saved them individually, and referenced them correctly according to the format requirements. | 16 |
| 7. We note that Figure 7 in your submission contain copyrighted images. All PLOS content is published under the Creative Commons Attribution License (CC BY 4.0), which means that the manuscript, images, and Supporting Information files will be freely available online, and any third party is permitted to access, download, copy, distribute, and use these materials in any way, even commercially, with proper attribution. | We have substituted the cartoon images in Figure 7 with original images that were drawn by the first author. The two original cartoon characters are portraits of the first author. Therefore, the copyright of the images belongs to the first author, and she agrees to publish them under the Creative Commons Attribution License (CC BY 4.0).     | -- |
| 8. Please review your reference list to ensure that it is complete and correct. If you have cited papers that have been retracted, please include the rationale for doing so in the manuscript text, or remove these references and replace them with relevant current references. Any changes to the reference list should be mentioned in the rebuttal letter that accompanies your revised manuscript. If you need to cite a retracted article, indicate the article’s retracted status in the References list and also include a citation and full reference for the retraction notice. | We have reviewed all the references to ensure their complete and correct. | 16-20 |
| Editor Comments | |  |
| 1. All the components (mainly introduction section) need to be concise and focused as they are currently too lengthy. Kindly condense them appropriately. | The whole paper including introduction, methods, discussion and references have been reduced from 7450 to 7100 words, especially, the introduction section has been reduced from 1460 to 1250 words. | -- |
| 2. The Discussion section requires further development. It should begin by providing a concise summary of the main findings, followed by situating them within the existing knowledge base, including relevant theories and empirical literature. Furthermore, it should comprehensively outline the implications of the findings for future research, policy-making, and practical applications. | We have modified the discussion section per your insightful suggestion. The first paragraph provides a concise summary of the project, and a comparison to existing literature; the second paragraph describes the strengths of this project; the third paragraph outlines the limitations; and the fourth paragraph discusses the implications of the findings. | 14-15 |
| 3. Please provide DOIs for all the references. | We have added DOIs for all the references. | 16-20 |
| Reviewers' Comments | |  |
| 1. Does the manuscript provide a valid rationale for the proposed study, with clearly identified and justified research questions?  Reviewer #1: Yes  Reviewer #2: Yes | -- | -- |
| 2. Is the protocol technically sound and planned in a manner that will lead to a meaningful outcome and allow testing the stated hypotheses?  Reviewer #1: Yes  Reviewer #2: Yes | -- | -- |
| 3. Is the methodology feasible and described in sufficient detail to allow the work to be replicable?   Reviewer #1: Yes  Reviewer #2: Yes | -- | -- |
| 4. Have the authors described where all data underlying the findings will be made available when the study is complete?   The PLOS Data policy [journals.plos.org] requires authors to make all data underlying the findings described in their manuscript fully available without restriction, with rare exception, at the time of publication. The data should be provided as part of the manuscript or its supporting information, or deposited to a public repository. For example, in addition to summary statistics, the data points behind means, medians and variance measures should be available. If there are restrictions on publicly sharing data—e.g. participant privacy or use of data from a third party—those must be specified.  Reviewer #1: Yes  Reviewer #2: No | We have added a description of the findings' implications and where to display them in the discussion section. | 15 |
| 5. Is the manuscript presented in an intelligible fashion and written in standard English?   Reviewer #1: Yes  Reviewer #2: Yes | -- | -- |
| Review Comments to the Author  Reviewer #1: Very well written, just a conclusion needs to be added explaining the future scope of research and how other scholars would benefit from this research. | Thank you so much for your valuable advice. We have referenced the publisher's recommendations for the protocol format and other publications in this journal, and then included the implications of the findings, the future research scope, and how other scholars can benefit from this study in the final paragraph. | 14-15 |
| Reviewer #2: I appreciate the opportunity to review your manuscript titled “Social-media based Health Education plus Exercise Programme (SHEEP) to improve muscle function among young-old adults with possible sarcopenia in the community a study protocol for intervention development". I would like to commend you for your efforts in researching this topic and for the valuable insights presented in the manuscript. Overall, I believe your study has the potential to make a significant contribution to the field of gerontology. However, there are a few areas that require attention and improvement. I have outlined my comments below (also specific suggestions and queries are annotated in a separate pdf file):  Your introduction provides a clear overview of the research problem and its significance. However, I recommend providing specific definition of possible sarcopenia with proper reference to make it more understandable to the readers. Also, I suggest to elaborate the existing literature that have cited, especially the population and area where the study have conducted to better contextualize the study. Emphasise on the previous studies that address social media-based intervention, digital health for older adults etc. This will strengthen the background and rationale for your research.  The ‘Methods and Analysis’ section is well structured, though some sub-sections required more elaboration to better understand your work as well as to replicate this protocol in similar future studies.  The discussion effectively summarizes the key aspects of this study and its requirement in recent decades. However, I encourage you to consider incorporating few relevant studies in the same vein to reflect your scope of plan with others.  Overall, I find your manuscript to be significant and worth considering for publication after addressing the suggestions. I believe that by incorporating these suggestions, your work will significantly enhance its clarity and impact on the field. | Thank you so much for your encouragement and patient guidance. We have carefully read each of your comments in the PDF file and made detailed modifications to the corresponding parts of the manuscript according to your suggestions.  Introduction   1. We have added the definition of possible sarcopenia at the beginning of the introduction section. 2. We have supplemented all cited studies with corresponding populations and regions in order to facilitate research status comparisons between regions. 3. We have detailed the findings from our scoping review that were instructive for this study. 4. We have listed several literatures to show that social media may be a promising medium for health education to disseminate knowledge and raise awareness of disease prevention among older adults.   Methods and Analysis   1. We have specified that the two rounds of focus groups will be conducted with the same participants, and that each round will consist of three groups, and that the participants will be distributed evenly among the three groups. 2. We have explained the reason that we contemplated setting the frequency of health education at 3 times/week. 3. To serve as a better resource for other scholars, we have added the initial multicomponent exercise strategy's detailed contents. 4. We have specified that if 80% of the total success criteria are met during pre-testing, the feasibility study will be initiated; otherwise, it will be modified to require an additional three weeks of pre-testing.   Discussion  On the basis of your advice, we have added several additional studies to elucidate current internet-based interventions for preventing sarcopenia in order to facilitate a more accurate comparison with this study. | 4  4  4-5  5  8  10  11  12  14-15 |

Finally, we really appreciate all the time and effort you put into this document, as well as all your insightful suggestions for revising the manuscript to make it more readable, clear, and academic. Thank you so much once more!

Best wishes
