# Supplementary material for: Social-media based Health Education plus Exercise Programme (SHEEP) to improve muscle function among community-dwelling young-old adults with possible sarcopenia in China: A study protocol for intervention development
Source: PLoS One. 2024 Mar 28;19(3):e0286490. doi: 10.1371/journal.pone.0286490 (PMC10977808; doi:10.1371/journal.pone.0286490)
Supplement: Attachment — Submitted filename: Response to Reviewers.docx [file pone.0286490.s007.docx]

**Response to comments**

**Dear Editor and Reviewers:**

Manuscript ID number: PONE-D-23-14790R1

Title: Social-media based Health Education plus Exercise Programme (SHEEP) to improve muscle function among young-old adults with possible sarcopenia in the community: a study protocol for intervention development

Thank you so much for your insightful feedback on our manuscript. We have revised and responded to each comment in accordance with your suggestion, as demonstrated below.

| **Journal requirements, editor comments and reviewers’ comments** | **Response** | **Page** |
| --- | --- | --- |
| Journal Requirements | |  |
| 1. Please review your reference list to ensure that it is complete and correct. If you have cited papers that have been retracted, please include the rationale for doing so in the manuscript text, or remove these references and replace them with relevant current references. Any changes to the reference list should be mentioned in the rebuttal letter that accompanies your revised manuscript. If you need to cite a retracted article, indicate the article’s retracted status in the References list and also include a citation and full reference for the retraction notice. | Thank you for this comment. We have reviewed and checked the reference list to ensure that it is complete and correct. | P  17-21 |
| Reviewers' Comments | |  |
| 1. Does the manuscript provide a valid rationale for the proposed study, with clearly identified and justified research questions?  Reviewer #2: Yes  Reviewer #3: Yes | --N/A | -- |
| 2. Is the protocol technically sound and planned in a manner that will lead to a meaningful outcome and allow testing the stated hypotheses?  Reviewer #2: Yes  Reviewer #3: Yes | --N/A | -- |
| 3. Is the methodology feasible and described in sufficient detail to allow the work to be replicable?   Reviewer #2: Yes  Reviewer #3: No | We have added more details in methodology section about how to develop the intervention strategy, according to the reviewers’ suggestion. | P12 |
| 4. Have the authors described where all data underlying the findings will be made available when the study is complete?   Reviewer #2: Yes  Reviewer #3: No | All of these findings will serve as evidence for our future feasibility study and will be disseminated via publication in peer-reviewed journals and presentation at relevant conferences, in order to serve as valuable resources, references, and directions for other researchers in related fields. | P16 |
| 5. Is the manuscript presented in an intelligible fashion and written in standard English?   Reviewer #2: Yes  Reviewer #3: Yes | --N/A | -- |
| 6. Review Comments to the Author   Reviewer #2: Appreciate your effort and dedication to addressing the comments/suggestions in improving this manuscript. The study intervention protocol is well described in methods section. Intervention studies in gerontology is utmost required to mitigate the burden of population ageing and to provide good health for older adults.  However, the manuscript required few minor corrections (highlighted in the pdf document). | Thank you so much for your valuable advice. We have carefully read each of your comments in the PDF file and made detailed modifications to the corresponding parts of the manuscript according to your suggestions.   1. We have included the year within parenthesis. 2. We have moved the definition of “young-old” in this study from the introduction to the methodology. 3. We have revised the format of the literature citation. 4. We have rewritten relevant sentences in the past tense in the section of study design. 5. We have revised the format of the literature citation. 6. We have deleted an extra comma “,”. 7. We have rewritten the word “pre-paring” as “preparing”. 8. We have deleted a repetition “to”. | P4  P4;P8  P5  P7  P8  P9  P10  P11 |
| Reviewer #3: This a a well written protocol. However, some points need clarification   a. Sample size calculation b. Literacy and social media use of the population studied, If the study protocol personnel want to know Tik-Tok use among the population, they could use a better topic, which would be more interesting   c. The setting is a population covered by a Nursing School - who are the content experts in this field who will vet the data and create the health education programs  d.Please clearly define which criteria you are going to use to define possible sarcopenia  e.Addressing possible sarcopenia is a multipronged approach. One intervention - that too unsupervised will not make much of a difference.  f. What are the end points - who will be the judge | Thank you so much for your encouragement and patient guidance. We have responded to your comments carefully.   a. Since this study was designed to develop an intervention strategy, there was no formula for sample size calculation. We carefully referred to other similar studies on intervention development to estimate sample size, and we also provided relevant references. b. Our goal is not to investigate the use of TikTok in the population. Our fundamental goal is to intervene in older adults with possible sarcopenia. Social media (TikTok) is just a tool for our intervention, and it is an attempt to combine the current trend of social media. The materials could be used in other social media platforms in the future if needed but our choice of TikTok for this study is due to its wide uptake and ability to engage with our study population. c. The Xiang Ya Nursing School we mentioned is our research partner, and they will provide the necessary experts, research equipment and site support for our study, but they do not participate in the management and analysis of the research data. During the preparing stage, the initial intervention plan was just designed by the research team based on literature reviews and team meetings.   We have added more details about how experts work in our study, as follows.  The development stage mainly includes two rounds of consultation with patient and public involvement (PPI) members and two rounds of focus groups to facilitate the development of an acceptable SHEEP intervention strategy for the targeted older population.  The first round of PPI consultation will be conducted face-to-face with six to eight young-old seniors between the ages of 60 and 69, who will be selected using convenience sampling methods. The purpose of this consultation is to gain preliminary understanding of the ability of Chinese young-old adults to understand and undertake the intervention; and to solicit their feedback on the content and duration of the initial SHEEP health education. Principal participants in the second round of PPI consultation will include a gerontologist, a geriatric nurse, a rehabilitation physician, and a rehabilitation nurse from Xiangya Nursing School and Xiangya Second Affiliated Hospital, Central South University, China. The gerontologist and geriatric nurse should have rich experience of geriatric health education, and their consultation is intended to make the SHEEP health education strategy more systematic, detailed, and in line with lay language requirements for Chinese older adults. Likewise, the purpose of the consultation with the rehabilitation physician and nurse with deep knowledge of geriatric sports medicine is to revise the SHEEP exercise intervention to ensure its safety and suitability for older people.   d. Because this study will be carried out in China, in the section of research participants, we have made it clear that the diagnostic criteria used in this study is the criteria for possible sarcopenia proposed by the Asian Working Group for Sarcopenia (AWGS, 2019). e. Our study is to co-design a multicomponent intervention (health education plus exercise) strategy for preventing possible sarcopenia through behaviour change in community-dwelling young-older adults. The purpose of health education is to improve the target population's understanding of the disease of sarcopenia and teach them ways to prevent sarcopenia, while the purpose of exercise is to help the targeted population to develop the habit of regular exercise to achieve long-term prevention of sarcopenia.   It is true that we cannot monitor the whole health education and exercise process of participants, which is the shortcoming of using social media for intervention. We will only conduct weekly follow-up by phone or text message, but in fact, this is one of the feasibility we will explore in this research program, that is, how feasible is using social media to carry out health education and exercise intervention? We will conduct a feasibility study in the next stage. The results of this study program can provide valuable reference for the subsequent research.   f. As this is a study for intervention development, no one will be judge at the end points. We will collect different opinions from different stakeholders about how to modify the intervention strategy, so the qualitative data from PPI consultation, focus groups and semi-structured interview will be the main results, which will further use to refine the intervention strategy. We (the study team) will use these findings to inform a future definitive study. | P8  P12  P8 |

Finally, we really appreciate all the time and effort you put into this document, as well as all your insightful suggestions for revising the manuscript to make it more readable, clear, and academic. Thank you so much once more!

Best wishes
