# Supplementary material for: Social-media based Health Education plus Exercise Programme (SHEEP) to improve muscle function among community-dwelling young-old adults with possible sarcopenia in China: A study protocol for intervention development
Source: PLoS One. 2024 Mar 28;19(3):e0286490. doi: 10.1371/journal.pone.0286490 (PMC10977808; doi:10.1371/journal.pone.0286490)
Supplement: Attachment — Submitted filename: 2024-01-18 Response to Reviewers.docx [file pone.0286490.s008.docx]

**Response to comments**

**Dear Editor and Reviewers:**

Manuscript ID number: PONE-D-23-14790R2

Title: Social-media based Health Education plus Exercise Programme (SHEEP) to improve muscle function among young-old adults with possible sarcopenia in the community: a study protocol for intervention development

Thank you so much for your insightful feedback on our manuscript. We have revised and responded to each comment in accordance with your suggestion, as demonstrated below.

| **Journal requirements, editor comments and reviewers’ comments** | **Response** | **Page** |
| --- | --- | --- |
| Journal Requirements | |  |
| 1. Please review your reference list to ensure that it is complete and correct. If you have cited papers that have been retracted, please include the rationale for doing so in the manuscript text, or remove these references and replace them with relevant current references. Any changes to the reference list should be mentioned in the rebuttal letter that accompanies your revised manuscript. If you need to cite a retracted article, indicate the article’s retracted status in the References list and also include a citation and full reference for the retraction notice. | We have reviewed and checked the reference list to ensure that it is complete and correct. | P  17-21 |
| Reviewers' Comments | |  |
| 1. Does the manuscript provide a valid rationale for the proposed study, with clearly identified and justified research questions?  Reviewer #4: Yes  Reviewer #5: Partly | We have added more evidence in the background section according to the suggestion from reviewer 5. | -- |
| 2. Is the protocol technically sound and planned in a manner that will lead to a meaningful outcome and allow testing the stated hypotheses?  Reviewer #4: Yes  Reviewer #5: Yes | -- | -- |
| 3. Is the methodology feasible and described in sufficient detail to allow the work to be replicable?   Reviewer #2: Yes  Reviewer #3: Yes | -- |  |
| 4. Have the authors described where all data underlying the findings will be made available when the study is complete?   Reviewer #4: Yes  Reviewer #5: No | All of these findings will serve as evidence for our future feasibility study and will be disseminated via publication in peer-reviewed journals and presentation at relevant conferences, and all relevant data will be made available within the manuscript and its Supporting Information files, in order to serve as valuable resources, references, and directions for other researchers in related fields. | P17 |
| 5. Is the manuscript presented in an intelligible fashion and written in standard English?   Reviewer #4: Yes  Reviewer #5: Yes | -- | -- |
| 6. Review Comments to the Author   Reviewer #4: The protocol has been well-written with sufficient and detailed description of the methodology. I look forward to the results of the study. | Thank you so much for your encouragement. |  |
| Reviewer #5: Comments:  I have read with interest the manuscript. The manuscript is well-written, and the findings are of utmost importance. Some minor comments and suggestions can be found below:  • Title page (page 9):  It may be worth to provide the name of the country where the intervention will be conducted.  • Abstract (page 11):  An option may be to define possible sarcopenia in the abstract.  • Keywords (page 11):  Keywords after the abstract are missing.  • Introduction; first paragraph (page 12):  Are there any data on the effects of possible sarcopenia on health and the economic burden of possible sarcopenia?  • Introduction; second paragraph (page 12):  How do the authors define young older adults?  • Introduction; third paragraph (page 12):  A formal definition of health education may be needed.  • Introduction; fourth paragraph (page 13):  Given that TikTok will be used for the intervention of interest, it is critical to provide more information on how TikTok has reached Chinese seniors in prior research.  • Introduction; sixth paragraph (page 14):  Has co-design already been used in the field of sarcopenia?  • Material and methods; Research participants (page 16):  The fact that the intervention will include young older adults already using TikTok may limit the generalizability of the findings of the study. Why have the authors not decided to provide some training to individuals not using TikTok prior to the intervention?  • Material and methods; Sampling method and sample size (page 16):  It is unclear to me why the authors decided to include 45 young older adults specifically.  • Material and methods; Research ethics (page 17):  It is critical to name the Chinese organizations of interest which granted permission for the study.  • Material and methods; Data collection and analysis (page 22):  How will the storage of the data be handled? Besides, in which country will the data be analyzed (e.g., China or the United Kingdom)? Finally, where all data underlying the findings will be made available when the study is complete?  • Discussion; first paragraph (page 23):  It could be interesting for the reader to provide the sample sizes of the different studies mentioned in this paragraph of the discussion.  • Discussion; fourth paragraph (page 24):  It may be worth considering providing more details on how the present intervention will shape future public health interventions and act as a platform for future research. | Thank you so much for your valuable advice and patient guidance. We have responded to your comments carefully.  We have clarified in the title that the study was conducted in China.  “Social-media based Health Education plus Exercise Programme (SHEEP) to improve muscle function among community-dwelling young-old adults with possible sarcopenia in China: a study protocol for intervention development”  Thank you for your advice. We have already described the definition of possible sarcopenia in the background section. We have also added the definition of possible sarcopenia in the abstract according to your suggestion.  Possible sarcopenia refers to low muscle strength.  Thank you for your advice. We have added key words in the abstract.  Key words: possible sarcopenia; older adults; community; social media; TikTok; health education; exercise; co-design; intervention development  Numerous evidence have shown that confirmed and severe sarcopenia is associated with a variety of adverse outcomes in older adults, such as increased risks of falls and fractures, functional decline, disability, lower quality of life, and higher risks of mortality. Untreated sarcopenia may result in significant personal, social, and economic burdens, which has been clarified in our previous scoping review [10]. Nevertheless, there is limited evidence regarding the impact of possible sarcopenia on health and the associated economic cost, warranting further investigation.  We have already clarified how we defined “young-old adults” in the section of “research participants”.  Although different scholars interpreted the definition of "young-old" differently, such as 60-69 years or 65-74 years [51,52], based on the fact that this study will be conducted in China, 60-69 years is considered young-old for the purposes of this study.  In fact, in the original version, we explained in detail why we chose 60-69 as young-old adults in this study. Due to the word limit, we simplified the description, and the detailed description is as follows.  Although age definition of an older person is not uniform to some extent around the world according to different conditions by different countries (e.g.≥50 in Africa, ≥60 in United Nations and China, ≥65 in western countries, ≥75 in Japan), the ages of 60 and 65 years are often used [A1-A3]. In addition, different scholars also have different interpretations of the definition of young-old, like 60-69 or 65-74 [A4-A5]. Based on the study shortage of 60-69 age group from my scoping review and also considering this feasible study will be carried out in China, 60-69 years old is defined as young-old in this study.  [A1] Kowal P, Dowd JE. Definition of an older person. Proposed working definition of an older person in Africa for the MDS Project. Geneva: World Health Organization 2001.  [A2] Orimo H, Ito H, Suzuki T, et al. Reviewing the definition of “elderly”. Geriatrics & Gerontology International 2006; 6(3): 149-158.  [A3] Ouchi Y, Rakugi H, Arai H, et al. Redefining the elderly as aged 75 years and older: Proposal from the Joint Committee of Japan Gerontological Society and the Japan Geriatrics Society. Geriatr Gerontol Int 2017; 17: 1045-1047.  [A4] Forman DE, Berman AD, McCabe CH, et al. PTCA in the elderly: The "young-old" versus the "old-old". Journal of the American Geriatrics Society 1992; 40 (1): 19-22.  [A5] Zizza CA, Ellison KJ, Wernette CM. Total Water Intakes of Community-Living Middle-Old and Oldest-Old Adults. The Journals of Gerontology Series A: Biological Sciences and Medical Sciences 2009; 64A (4): 481-486.  Thank you for your advice. We have added the definition of health education.  Health Education, as defined by the World Health Organization, refers to the deliberate creation of learning opportunities that involve communication aimed at enhancing health literacy. This includes improving knowledge and developing life skills that promote individual and community health [11].  We have added more references about how TikTok has reached older adults on health information.  A qualitative research showed that during the COVID-19 pandemic, WeChat and TikTok played a significant role in providing older Chinese adults with access to valuable health information; and many older adults were able to modify their health behaviours after incorporating this information and knowledge into their daily lives [27]. Kassamali et al. [28] reported that TikTok held great potential as a platform for disseminating educational information about disease, as their study indicated that the number of views for the hashtag such as #acne, #alopecia, #cyst, #rosacea, and #psoriasis on TikTok doubled in just 5 months. Nonetheless, there are currently no studies utilizing social media for sarcopenia prevention.  We have added a sentence in the sixth paragraph as following:  “There is currently no evidence of co-design in the field of sarcopenia research, making the exploration of this study particularly valuable.”  Insufficient time during the intervention development phase prevented us from adequately training a cohort of older individuals who were unfamiliar with TiKTok, which may be a limitation of this study. We also mentioned this in the limitations section of this study. However, our forthcoming feasibility study will offer training to older persons who are eager to utilize TikTok before the intervention, in order to expand the range of recruitment.  Older adults who are unfamiliar with TikTok or lack Internet and modern communication technology access or proficiency were excluded from the recruitment stage, which may result in a less diverse sample.  We have clarified clearly in the section of “sample size”. 45 is the maximum number of recruits and is made up of two parts. The sample size was calculated with reference to similar studies about focus group and co-design research.  We aim to recruit a maximum sample of 45 older adults in total. For developing phase, 18~25 older adults will be recruited to participate in focus groups (with a 10~15% attrition rate), and then invited to take part in both the first and the second round of focus groups. Each round will consist of three focus groups, with 18~25 participants divided into three groups of at least six members each, which is based on the recommendations of Leask et al.[50] that 10~12 co-creators in total is advised in co-design research and the Guidelines for Conducting a Focus Group that suggests six to ten people will be appropriate for a focus group [55]. Besides, we require 15~20 additional participants for the pre-test phase. In the two phases of our study, participants have the option of participating in only focus groups, only pre-test, or both focus groups and pre-test.  We have added the name of the Chinese organization.  This study has been approved by the University of Manchester Research Ethics Committee (Project ID: 15664), and permissions have already been granted by collaborators in the Community Nursing Department of Xiang Ya Nursing School, Central South University, China.  We have clarified this part as following:  All data will be saved on servers or computers that are certified by the University of Manchester and have safe backup systems. The data will be analyzed in China, in accordance with the data management plan.  All of these findings will serve as evidence for our future feasibility study and will be disseminated via publication in peer-reviewed journals and presentation at relevant conferences, and all relevant data will be made available within the manuscript and its Supporting Information files, in order to serve as valuable resources, references, and directions for other researchers in related fields.  We have added the sample sizes of the different studies mentioned in this paragraph of the discussion.  One study in China involved a 12-week intervention of 234 older adults with sarcopenia and revealed that nutrition interventions through an app were effective in optimising the diets and increasing the skeletal muscle mass [63]. Another study conducted a randomized controlled trial on 23 participants and indicated that supervised resistance exercise through a video conferencing software (Skype™) had positive effects on sarcopenia-related factors among community-dwelling senior citizens in South Korea [64].  We have added more details according to your suggestion as following:  Furthermore, the comprehensive co-design procedure will build a foundation for co-design research in the field of sarcopenia. Additionally, the health education and exercise video resources on TikTok will serve as valuable reference materials for future intervention by other researchers. | P1,3  P3  P3  --  P8  P4  P5-6  P6-7  P16  P8-9  P10  P14  P17  P16  P17 |

Finally, we really appreciate all the time and effort you put into this document, as well as all your insightful suggestions for revising the manuscript to make it more readable, clear, and academic. Thank you so much once more!

Best wishes
